# Supplementary material for: End Sequence Analysis Toolkit (ESAT) expands the extractable information from single-cell RNA-seq data
Source: Genome Res. 2016 Oct;26(10):1397–410. doi: 10.1101/gr.207902.116 (PMC5052061; doi:10.1101/gr.207902.116)
Supplement: Supplemental Material [file supp_gr.207902.116_Supp_Materials.tar.gz › ESAT-esat_v0.1/doc/html/classbroad_1_1core_1_1siphy_1_1_evolutionary_model.html]

ESAT: broad.core.siphy.EvolutionaryModel Class Reference


ESAT


- Main Page
- Related Pages
- Packages
- Classes
- Files

- Class List
- Class Index
- Class Hierarchy
- Class Members

All Classes Namespaces Files Functions Variables Enumerator Pages

- broad
- core
- siphy
- EvolutionaryModel

Classes |
Public Member Functions |
Protected Member Functions |
List of all members

broad.core.siphy.EvolutionaryModel Class Reference

Collaboration diagram for broad.core.siphy.EvolutionaryModel:

[legend]

|  |  |
| --- | --- |
| Classes | |
| class | **NodeLikelihoodParameters** |
|  | |
| class | **OmegaFit** |
|  | |
| class | **PiFit** |
|  | |

|  |  |
| --- | --- |
| Public Member Functions | |
|  | EvolutionaryModel (EvolutionaryModelParameters parameters) |
|  | |
| void | setOmega (double omega) |
|  | |
| void | RPIDecomposition () |
|  | |
| Map< String, Short > | sample () |
|  | |
| Map< String, Short > | sample (PhylogenyNode node, short parentState) |
|  | |
| PiFit | fitPI (Map< String, Matrix > alignmentColumn, Phylogeny tree) |
|  | |
| OmegaFit | fitOmega (Map< String, Matrix > leafValues, Phylogeny tree, int window) |
|  | |
| PiFit | piEMIteration (Map< String, Matrix > column, PhylogenyNode root, Matrix newPI) throws UnableToFitException |
|  | |
| double[] | omegaEMIteration (Map< String, Matrix > leafValues, PhylogenyNode root, double newOmega, int window) |
|  | |
| double | computeLikelihood (Map< String, Matrix > leafValues, PhylogenyNode root, int site) |
|  | |
| double | pruneAndPeel (Map< String, Matrix > leafValues, PhylogenyNode root, int site) |
|  | |
| void | computeJMatrix (PhylogenyNode node) |
|  | |
| Map< Integer,   NodeLikelihoodParameters > | getNodeLikelihoodParameterMap () |
|  | |
| Matrix | computeTransitions (double branchLength) |
|  | |
| void | setModelParameters (EvolutionaryModelParameters parameters) throws IllegalArgumentException |
|  | |
| EvolutionaryModelParameters | getModelParameters () |
|  | |
| Phylogeny | getTree () |
|  | |
| void | setOmegaDistByTreeLength (String neutralOmegaDistributionByTreeLengthFile) throws IOException |
|  | |
| double | getPVal (OmegaFit fit, boolean useLogOddsLikelihood) |
|  | |
| void | setNeutralOmegaDistribution (String neutralOmegaFile) throws IOException |
|  | |
| void | resetPI () |
|  | |
| EvolutionaryModel | copy () |
|  | |
| EvolutionaryModelParameters | getParameters () |
|  | |
| void | setParameters (EvolutionaryModelParameters parameters) |
|  | |
| int | getAlphabetSize () |
|  | |
| void | setAlphabetSize (int alphabetSize) |
|  | |
| Matrix | getPi () |
|  | |
| void | setPi (Matrix pi) |
|  | |
| void | pruneTree (List< String > ignoreList) |
|  | |
| Phylogeny | evolve (String sequence) |
|  | |
| void | changeBackground (double pA, double pC, double pG, double pT) |
|  | |
| void | changeBackground (Matrix newPi) |
|  | |

|  |  |
| --- | --- |
| Protected Member Functions | |
| Matrix | getRateMatrix () |
|  | |

## Detailed Description

Represents an Markovian Evolutionary Model

Author
:   mgarber

## Constructor & Destructor Documentation

|  |  |  |  |  |  |
| --- | --- | --- | --- | --- | --- |
| broad.core.siphy.EvolutionaryModel.EvolutionaryModel | ( | EvolutionaryModelParameters | *parameters* | ) |  |

Here is the call graph for this function:

Here is the caller graph for this function:

## Member Function Documentation

|  |  |  |  |
| --- | --- | --- | --- |
| void broad.core.siphy.EvolutionaryModel.changeBackground | ( | double | *pA*, |
|  |  | double | *pC*, |
|  |  | double | *pG*, |
|  |  | double | *pT* |
|  | ) |  |  |

|  |  |  |  |  |  |
| --- | --- | --- | --- | --- | --- |
| void broad.core.siphy.EvolutionaryModel.changeBackground | ( | Matrix | *newPi* | ) |  |

Here is the call graph for this function:

|  |  |  |  |  |  |
| --- | --- | --- | --- | --- | --- |
| void broad.core.siphy.EvolutionaryModel.computeJMatrix | ( | PhylogenyNode | *node* | ) |  |

|  |  |  |  |
| --- | --- | --- | --- |
| double broad.core.siphy.EvolutionaryModel.computeLikelihood | ( | Map< String, Matrix > | *leafValues*, |
|  |  | PhylogenyNode | *root*, |
|  |  | int | *site* |
|  | ) |  |  |

Here is the call graph for this function:

Here is the caller graph for this function:

|  |  |  |  |  |  |
| --- | --- | --- | --- | --- | --- |
| Matrix broad.core.siphy.EvolutionaryModel.computeTransitions | ( | double | *branchLength* | ) |  |

Computes the vector of transition probabilities for the given branch length and value of the end state.

Parameters
:   |  |  |
    | --- | --- |
    | branchLenght | distance to the end state. |

Returns
:   P the transition probabilities matrix for the given branch length.

Here is the caller graph for this function:

|  |  |  |  |  |
| --- | --- | --- | --- | --- |
| EvolutionaryModel broad.core.siphy.EvolutionaryModel.copy | ( |  | ) |  |

Here is the call graph for this function:

Here is the caller graph for this function:

|  |  |  |  |  |  |
| --- | --- | --- | --- | --- | --- |
| Phylogeny broad.core.siphy.EvolutionaryModel.evolve | ( | String | *sequence* | ) |  |

Assumes the given sequence as ancestral, the runs the markov process forward to generate sequences at ach node.

Parameters
:   |  |  |
    | --- | --- |
    | sequence | - ancestral sequence |

Returns
:   Phylogeny - with the same topology as the model's but its node names have been replaced with the evolved sequence.

Here is the call graph for this function:

|  |  |  |  |
| --- | --- | --- | --- |
| OmegaFit broad.core.siphy.EvolutionaryModel.fitOmega | ( | Map< String, Matrix > | *leafValues*, |
|  |  | Phylogeny | *tree*, |
|  |  | int | *window* |
|  | ) |  |  |

Implementation of Felsenstein's prunning algorithm to compute the likelihood given this model.

Parameters
:   |  |  |
    | --- | --- |
    | observedData |  |

Returns

Exceptions
:   |  |  |
    | --- | --- |
    | MathException |  |

Here is the call graph for this function:

|  |  |  |  |
| --- | --- | --- | --- |
| PiFit broad.core.siphy.EvolutionaryModel.fitPI | ( | Map< String, Matrix > | *alignmentColumn*, |
|  |  | Phylogeny | *tree* |
|  | ) |  |  |

Implementation of the EM algorithm to fit the base distribution rho at a site assuming omega is constant.

Exceptions
:   |  |  |
    | --- | --- |
    | UnableToFitException |  |

Here is the call graph for this function:

|  |  |  |  |  |
| --- | --- | --- | --- | --- |
| int broad.core.siphy.EvolutionaryModel.getAlphabetSize | ( |  | ) |  |

Here is the caller graph for this function:

|  |  |  |  |  |
| --- | --- | --- | --- | --- |
| EvolutionaryModelParameters broad.core.siphy.EvolutionaryModel.getModelParameters | ( |  | ) |  |

|  |  |  |  |  |
| --- | --- | --- | --- | --- |
| Map<Integer,NodeLikelihoodParameters> broad.core.siphy.EvolutionaryModel.getNodeLikelihoodParameterMap | ( |  | ) |  |

|  |  |  |  |  |
| --- | --- | --- | --- | --- |
| EvolutionaryModelParameters broad.core.siphy.EvolutionaryModel.getParameters | ( |  | ) |  |

|  |  |  |  |  |
| --- | --- | --- | --- | --- |
| Matrix broad.core.siphy.EvolutionaryModel.getPi | ( |  | ) |  |

|  |  |  |  |
| --- | --- | --- | --- |
| double broad.core.siphy.EvolutionaryModel.getPVal | ( | OmegaFit | *fit*, |
|  |  | boolean | *useLogOddsLikelihood* |
|  | ) |  |  |

|  |  |  |  |  |
| --- | --- | --- | --- | --- |
| Matrix broad.core.siphy.EvolutionaryModel.getRateMatrix | ( |  | ) |  |

protected

Computes current model's rate matrix. If the model is Q = R\*rho then it computes Q, otherwise it returns the rate matrix in the Model Parameters

Returns
:   An update rate matrix.

Here is the caller graph for this function:

|  |  |  |  |  |
| --- | --- | --- | --- | --- |
| Phylogeny broad.core.siphy.EvolutionaryModel.getTree | ( |  | ) |  |

|  |  |  |  |
| --- | --- | --- | --- |
| double [] broad.core.siphy.EvolutionaryModel.omegaEMIteration | ( | Map< String, Matrix > | *leafValues*, |
|  |  | PhylogenyNode | *root*, |
|  |  | double | *newOmega*, |
|  |  | int | *window* |
|  | ) |  |  |

Here is the call graph for this function:

Here is the caller graph for this function:

|  |  |  |  |
| --- | --- | --- | --- |
| PiFit broad.core.siphy.EvolutionaryModel.piEMIteration | ( | Map< String, Matrix > | *column*, |
|  |  | PhylogenyNode | *root*, |
|  |  | Matrix | *newPI* |
|  | ) |  | throws UnableToFitException |

Here is the call graph for this function:

Here is the caller graph for this function:

|  |  |  |  |
| --- | --- | --- | --- |
| double broad.core.siphy.EvolutionaryModel.pruneAndPeel | ( | Map< String, Matrix > | *leafValues*, |
|  |  | PhylogenyNode | *root*, |
|  |  | int | *site* |
|  | ) |  |  |

Here is the call graph for this function:

Here is the caller graph for this function:

|  |  |  |  |  |  |
| --- | --- | --- | --- | --- | --- |
| void broad.core.siphy.EvolutionaryModel.pruneTree | ( | List< String > | *ignoreList* | ) |  |

|  |  |  |  |  |
| --- | --- | --- | --- | --- |
| void broad.core.siphy.EvolutionaryModel.resetPI | ( |  | ) |  |

Here is the call graph for this function:

Here is the caller graph for this function:

|  |  |  |  |  |
| --- | --- | --- | --- | --- |
| void broad.core.siphy.EvolutionaryModel.RPIDecomposition | ( |  | ) |  |

Here is the caller graph for this function:

|  |  |  |  |  |
| --- | --- | --- | --- | --- |
| Map<String, Short> broad.core.siphy.EvolutionaryModel.sample | ( |  | ) |  |

Here is the caller graph for this function:

|  |  |  |  |
| --- | --- | --- | --- |
| Map<String,Short> broad.core.siphy.EvolutionaryModel.sample | ( | PhylogenyNode | *node*, |
|  |  | short | *parentState* |
|  | ) |  |  |

Here is the call graph for this function:

|  |  |  |  |  |  |
| --- | --- | --- | --- | --- | --- |
| void broad.core.siphy.EvolutionaryModel.setAlphabetSize | ( | int | *alphabetSize* | ) |  |

|  |  |  |  |  |  |
| --- | --- | --- | --- | --- | --- |
| void broad.core.siphy.EvolutionaryModel.setModelParameters | ( | EvolutionaryModelParameters | *parameters* | ) | throws IllegalArgumentException |

Here is the caller graph for this function:

|  |  |  |  |  |  |
| --- | --- | --- | --- | --- | --- |
| void broad.core.siphy.EvolutionaryModel.setNeutralOmegaDistribution | ( | String | *neutralOmegaFile* | ) | throws IOException |

|  |  |  |  |  |  |
| --- | --- | --- | --- | --- | --- |
| void broad.core.siphy.EvolutionaryModel.setOmega | ( | double | *omega* | ) |  |

Here is the caller graph for this function:

|  |  |  |  |  |  |
| --- | --- | --- | --- | --- | --- |
| void broad.core.siphy.EvolutionaryModel.setOmegaDistByTreeLength | ( | String | *neutralOmegaDistributionByTreeLengthFile* | ) | throws IOException |

|  |  |  |  |  |  |
| --- | --- | --- | --- | --- | --- |
| void broad.core.siphy.EvolutionaryModel.setParameters | ( | EvolutionaryModelParameters | *parameters* | ) |  |

|  |  |  |  |  |  |
| --- | --- | --- | --- | --- | --- |
| void broad.core.siphy.EvolutionaryModel.setPi | ( | Matrix | *pi* | ) |  |

Here is the call graph for this function:

Here is the caller graph for this function:

---

The documentation for this class was generated from the following file:

- java/broad/core/siphy/EvolutionaryModel.java


---

Generated on Fri Aug 15 2014 15:10:57 for ESAT by  

 1.8.7
